# Accuracy of the clinical pulmonary infection score to differentiate ventilator-associated tracheobronchitis from ventilator-associated pneumonia

**DOI:** 10.1186/s13613-020-00721-4

**Published:** 2020-08-03

**Authors:** Alexandre Gaudet, Ignacio Martin-Loeches, Pedro Povoa, Alejandro Rodriguez, Jorge Salluh, Alain Duhamel, Saad Nseir, TAVeM study group

**Affiliations:** 1 Department of Intensive Care Medicine, Critical Care Centre, CHU Lille, Lille, 59000 France; 2 Univ. Lille, CNRS, Inserm, CHU Lille, Institut Pasteur de Lille, U1019-UMR9017-CIIL-Centre d’Infection et d’Immunité de Lille, Lille, France; 3 Department of Intensive Care Medicine, Multidisciplinary Intensive Care Research Organization (MICRO), St. James’s Hospital, St James Street, Dublin 8, Dublin, Ireland; 4 Hospital Clinic, IDIBAPS, Universidad de Barcelona, Ciberes, Barcelona, Spain; 5 Polyvalent Intensive Care Unit, São Francisco Xavier Hospital, Centro Hospitalar de Lisboa Ocidental, Lisbon, Portugal; 6 NOVA Medical School, New University of Lisbon, Lisbon, Portugal; 7 Hospital Universitari Joan XXIII, Critical Care Medicine, Rovira & Virgili University, Rovira, Tarragona, Spain; 8 Centro de Investigación Biomédica en Red de Enfermedades Respiratorias, Instituto de Salud Carlos III, Bunyola, Mallorca Spain; 9 Department of Critical Care and Graduate Program in Translational Medicine, Programa de Pós-Graduação em Clínica Médica, D’Or Institute for Research and Education, Rio de Janeiro, Brazil; 10 Univ. Lille, CHU Lille, ULR 2694 METRICS- Evaluation des technologies de santé et des pratiques médicales, 59000 Lille, France; 11 CHU Lille, Unité de Méthodologie, Biostatistiques et Data Management, Lille, 59000 France; 12 Université de Lille, INSERM U995, Lille Inflammation Research International Center E2, Lille, 59000 France

**Keywords:** Mechanical ventilation, Lower respiratory tract infection, Tracheobronchitis, Pneumonia, CPIS

## Abstract

**Background:**

Differentiating Ventilator-Associated Tracheobronchitis (VAT) from Ventilator-Associated Pneumonia (VAP) may be challenging for clinicians, yet their management currently differs. In this study, we evaluated the accuracy of the Clinical Pulmonary Infection Score (CPIS) to differentiate VAT and VAP.

**Methods:**

We performed a retrospective analysis based on the data from 2 independent prospective cohorts. Patients of the TAVeM database with a diagnosis of VAT (*n* = 320) or VAP (*n* = 369) were included in the derivation cohort. Patients admitted to the Intensive Care Centre of Lille University Hospital between January 1, 2016 and December 31, 2017 who had a diagnosis of VAT (*n* = 70) or VAP (*n* = 139) were included in the validation cohort. The accuracy of the CPIS to differentiate VAT from VAP was assessed within the 2 cohorts by calculating sensitivity and specificity values, establishing the ROC curves and choosing the best threshold according to the Youden index.

**Results:**

The areas under ROC curves of CPIS to differentiate VAT from VAP were calculated at 0.76 (95% CI [0.72–0.79]) in the derivation cohort and 0.67 (95% CI [0.6–0.75]) in the validation cohort. A CPIS value ≥ 7 was associated with the highest Youden index in both cohorts. With this cut-off, sensitivity and specificity were respectively found at 0.51 and 0.88 in the derivation cohort, and at 0.45 and 0.89 in the validation cohort.

**Conclusions:**

A CPIS value ≥ 7 reproducibly allowed to differentiate VAT from VAP with high specificity and PPV and moderate sensitivity and NPV in our derivation and validation cohorts.

## Background

Ventilator-Associated Lower Respiratory Tract Infections (VA-LRTI), including Ventilator-Associated Tracheobronchitis (VAT) and Ventilator-Associated Pneumonia (VAP), are the most frequent infectious complications in Intensive Care Units (ICU), concerning about 25% of critically ill subjects undergoing mechanical ventilation [1]. Their diagnosis currently relies on symptoms of lower respiratory tract infection in patients intubated for more than 48 h with a positive culture of lower respiratory microbiological sampling. In addition to these criteria, the presence of a new infiltrate on chest radiography allows to make the diagnosis of VAP. By contrast, VAT is characterized by the combination of the above-mentioned criteria without new radiographic infiltrates [2].

The occurrence of VAP is associated with increased mortality, longer duration of mechanical ventilation and length of stay in the ICU. On the other hand, the diagnosis of VAT seems to be linked with lower levels of mortality than VAP, even though being associated with increased length of mechanical ventilation and ICU stay [1]. The management of VAP currently relies on antimicrobial therapy, whereas current guidelines do not recommend the administration of antibiotics in VAT, making the distinction between these two entities a crucial point [2].

Although theoretically based on clearly defined criteria, differentiating VAT and VAP may sometimes be challenging for the physician. Indeed, chest radiographies performed in the context of ICU often lead to multiple artefacts, additionally to numerous causes of non-infectious radiological opacities making the diagnosis of VAP tricky. In addition, a lack of sensitivity of chest radiography has been reported for the detection of VAP, thus leading to a likely underestimation of this diagnosis in mechanically ventilated patients [3–5].

Several tools have been developed to improve the detection of VAP. Amongst them, the Clinical Pulmonary Infection Score (CPIS) is a daily routine parameter-based score with moderate to good accuracy in the detection of VAP. This score, originally described by Pugin et al, has been declined in a simplified version, allowing its easier appliance by physicians at patient’s bedside [6, 7]. Based on the results of a single study, recent guidelines on the management of Hospital-Acquired Pneumonia and VAP suggest that a CPIS score ≤ 6 should lead to the early discontinuation of antimicrobial therapy, being associated with a low probability of pneumonia [2, 8].

Considering these observations, the use of the CPIS in patients with microbiologically confirmed VA-LRTI might be proposed as a helpful tool for the early detection of VAP. However, to the best of our knowledge, the evaluation of the CPIS in this indication has never been reported yet and would be of significant interest. Furthermore, such an evaluation should be preferentially performed in 2 independent cohorts to assess its reproducibility, given the heterogeneity in the performances of CPIS for the diagnosis of VAP in ventilated patients [9], and because this score was not initially developed to distinguish VAT from VAP.

Therefore, we aimed in this study to evaluate the accuracy of the CPIS to differentiate VAT from VAP in 2 independent cohorts of patients with microbiologically confirmed VA-LRTI.

## Methods

### Study design and patients

This is a retrospective study based on the analysis from the TAVeM database [1] and of a cohort of patients admitted in a single mixed ICU during a 2-year period.

The TAVeM study is a large prospective multinational observational study conducted in 114 ICUs in Europe and South America. Details about the design and patients of the TAVeM study have been previously published [1].

In our study, patients from the TAVeM database with a diagnosis of VA-LRTI were included in the derivation cohort.

Patients admitted in the single mixed ICU of the Lille University Hospital between 1 January 2016 and 31 December 2017 and with a diagnosis of VA-LRTI were included in the validation cohort.

### Data collection

Patient demographic characteristics, severity scores, comorbidities, primary diagnoses, and prior antibiotic exposure were recorded at baseline for all patients. Further, data about clinical, biological and radiological diagnostic criteria for VA-LRTI, microbiological diagnostic procedures, bacteriological findings, degree of severity on the onset of infection, antibiotic use and clinical outcomes were obtained.

### Definitions

#### VA-LRTI

The diagnosis of VA-LRTI was based on the presence of at least 2 of the following criteria: body temperature of more than 38.5 °C or less than 36.5 °C, leucocyte count greater than 12,000 cells per μL or less than 4 000 cells per μL, and purulent endotracheal aspirate. Microbiological confirmation was needed for all episodes of infection, with the isolation in the endotracheal aspirate of at least 10^5^ CFU per mL, or in bronchoalveolar lavage of at least 10^4^ CFU per mL.

VAT was defined with the above-mentioned criteria with no radiographical signs of new pneumonia. Conversely, VAP was defined by the presence of new or progressive infiltrates on chest radiograph.

#### CPIS

A modified version of the CPIS was used in this study, as previously published in the literature (Additional file 1) [7]. Notably, we did not consider cultures of tracheal aspirate; leucocyte categories were reduced to two; three categories were used for the aspect of sputum; tracheal secretions were classified as few, moderate, large, and purulent. The CPIS score was calculated at the time of microbiological sampling for clinical suspicion of VA-LRTI by retaining for each variable the maximal attributable number of points over the 24 past hours.

#### Outcomes

The primary aim of this study was to evaluate the accuracy of the CPIS to differentiate VAT from VAP in patients with microbiologically confirmed VA-LRTI.

### Statistical analysis

Categorical variables were expressed as numbers (percentages) and compared using Chi square test or Fisher’s exact test, as appropriate. Normality of distribution of continuous variables was checked graphically and using the Shapiro–Wilk test. Skewed continuous variables were presented as medians (interquartile ranges), and compared using Mann–Whitney *U* tests. Normally distributed continuous variables were presented as means (SD), and compared using Student’s *t* tests. Correlations between skewed continuous variables were assessed using Spearman’s rank correlation tests, and regression lines were displayed using simple linear regression method.

To assess the diagnostic ability of CPIS to differentiate VAT from VAP, we performed a Receiver Operating Characteristics (ROC) analysis for the diagnosis of VAP in both derivation and validation cohorts. We computed the area under the ROC curve (AUC) and the sensitivity, specificity, positive and negative predictive values (PPV and NPV) as well as positive and negative likelihood ratios for different cut-off values. The best cut-off for the discrimination between VAP and VAT was determined according to the Youden index.

All statistical tests were two-tailed and *p* values < 0.05 were considered statistically significant. Statistics Department of Lille University Hospital performed all data analyses using SAS software package, release 9.4 (SAS Institute, Cary, NC, USA).

## Results

Three hundred twenty patients with VAT and 369 patients with VAP were included in the derivation cohort. Seventy patients with VAT and 136 patients with VAP were included in the validation cohort.

Percentage of male and percentage of cirrhosis were higher, whilst age was lower in patients with VAP compared to patients with VAT in the derivation cohort. SOFA score was higher and percentage of heart failure was lower in patients with VAP compared to patients with VAT in the validation cohort. Appropriate initial antimicrobial therapy was more frequent in VAP than in VAT in the derivation cohort, and less frequent in VAP compared to VAT in the validation cohort (Table 1). Comparisons of baseline patients characteristics between derivation and validation cohorts are shown in Additional file 2.

**Table 1:** Baseline characteristics of patients

	Derivation cohort		P value	Validation cohort		*P* value
	VAT (*n* = 320)	VAP (*n* = 369)		VAT (*n* = 70)	VAP (*n* = 136)	
Sex						
Male	199 (62%)	264 (72%)		51 (73%)	102 (75%)	
Female	121 (38%)	105 (28%)	*0.009*	19 (27%)	34 (25%)	0.74
Age (years)	61.20 (16.25)	57.74 (18.46)	*0.009*	55.26 (16.1)	54.98 (16.36)	0.89
SAPS II	48.85 (18.12)	49.89 (17.80)	0.45	54.6 (19.46)	59.97 (16.68)	0.079
SOFA	7.64 (3.79)	8.07 (3.63)	0.13	7.19 (4.34)	8.9 (4.04)	*0.008*
Admission category						
Medical	175 (55%)	218 (59%)		57 (81%)	117 (86%)	
Surgical	145 (45%)	151 (41%)	0.25	13 (19%)	19 (14%)	0.39
COPD	64 (20%)	61 (17%)	0.24	6 (10%)	21 (15%)	0.17
Diabetes mellitus	66 (21%)	69 (19%)	0.53	14 (20%)	26 (20%)	0.88
Immunocompromised patients	24 (8%)	31 (8%)	0.66	7 (10%)	23 (17%)	0.18
Chronic heart failure	27 (8%)	19 (5%)	0.085	16 (23%)	16 (12%)	*0.037*
Chronic respiratory failure	35 (11%)	27 (7%)	0.098	3 (4%)	7 (5%)	0.78
Cirrhosis	8 (2%)	23 (6%)	*0.018*	3 (4%)	10 (7%)	0.38
Previous antibiotic use	210 (66%)	232 (63%)	0.45	60 (86%)	107 (79%)	0.3
Appropriate initial antimicrobial therapy	174 (54%)	257 (70%)	< 0.001	40 (57%)	55 (40%)	0.023

Data are presented as number (%) or mean (SD)

*COPD* Chronic obstructive pulmonary disease, *SAPS* Simplified acute physiology score, *SOFA* Sequential organ failure assessment, *VAP* Ventilator-associated pneumonia, *VAT* Ventilator-associated tracheobronchitis

*p* value < 0.05 is indicated in italic characters

Clinical outcomes in the derivation and validation cohorts are shown in Table 2 and Additional file 3. ICU mortality was higher in patients with VAP compared to patients with VAT in the derivation cohort.

**Table 2:** Clinical outcomes of patients

	Derivation cohort		*P* value	Validation cohort		*P* value
	VAT (*n* = 320)	VAP (*n* = 369)		VAT (*n* = 70)	VAP (*n* = 136)	
Days on mechanical ventilation	13 (8–20)	13 (8–26)	0.35	18 (13–29)	17 (11–29)	0.23
Days in the ICU	21 (15–34)	22 (13–36)	0.69	24 (16 – 37)	22 (14–35)	0.3
ICU mortality	93 (29%)	146 (40%)	*0.004*	18 (26%)	46 (34%)	0.23
Progression from VAT to VAP	39 (12%)	–	**–**	7 (10%)	–	–

Data are presented as number (%) or median (interquartile range)

*ICU* Intensive Care Unit; *VAP* Ventilator-Associated Pneumonia; *VAT* Ventilator-Associated Tracheobronchitis

*p* values < 0.05 are indicated in italics characters

The analysis of microbiological findings revealed a higher frequency of *Enterobacter* spp. in patients with VAP compared to patients with VAT in the validation cohort. Conversely, *Citrobacter freundii* was found more frequently in VATs than in VAPs in the validation cohort (Table 3). Comparisons of microbiological findings between derivation and validation cohorts are shown in Additional file 4. CPIS values ranged from 0 to 10 for patients with VAT and from 0 to 11 for patients with VAP in the derivation cohort. In the validation cohort, CPIS values ranged from 2 to 10 for patients with VAT and from 1 to 10 for patients with VAP.

**Table 3:** Microbiological findings

	Derivation cohort		P value	Validation cohort		*P* value
	VAT (*n* = 320)	VAP (*n* = 369)		VAT (*n* = 70)	VAP (*n* = 136)	
*Streptococcus pneumoniae*	16 (5%)	24 (7%)	0.41	2 (3%)	5 (4%)	1
*Stenotrophomonas maltophila*	19 (6%)	12 (3%)	0.09	4 (6%)	6 (4%)	0.74
MRSA	8 (2%)	8 (2%)	0.77	1 (1%)	1 (1%)	1
MSSA	66 (21%)	80 (22%)	0.73	6 (9%)	21 (15%)	0.17
*Serratia marcescens*	12 (4%)	16 (4%)	0.69	3 (4%)	5 (4%)	1
*Pseudomonas aeruginosa*	79 (25%)	89 (24%)	0.86	20 (29%)	29 (21%)	0.25
*Proteus mirabilis*	15 (5%)	14 (4%)	0.56	3 (4%)	5 (4%)	1
*Klebsiella pneumoniae*	48 (15%)	53 (14%)	0.81	9 (13%)	30 (22%)	0.11
*Haemophilus influenzae*	32 (10%)	25 (7%)	0.12	4 (6%)	6 (4%)	0.74
*Escherichia coli*	37 (12%)	40 (11%)	0.76	5 (7%)	9 (7%)	1
*Enterobacter* spp.	35 (11%)	46 (12%)	0.53	4 (6%)	21 (15%)	*0.043*
*Citrobacter freundii*	7 (2%)	6 (2%)	0.58	4 (6%)	1 (1%)	*0.047*
*Acinetobacter baumannii*	14 (4%)	27 (7%)	0.10	0 (0%)	2 (1%)	0.55

Data are presented as number (%) or mean (SD)

*COPD* Chronic obstructive pulmonary disease, *MSSA* Methicillin-sensitive *Staphylococcus Aureus*, *MRSA* Methicillin-resistant *Staphylococcus Aureus, SAPS* Simplified acute physiology score, *SOFA* Sequential organ failure assessment, *VAP* Ventilator-associated pneumonia, *VAT* Ventilator-associated tracheobronchitis

*p* values < 0.05 are indicated in italic characters

**Fig. 1:**
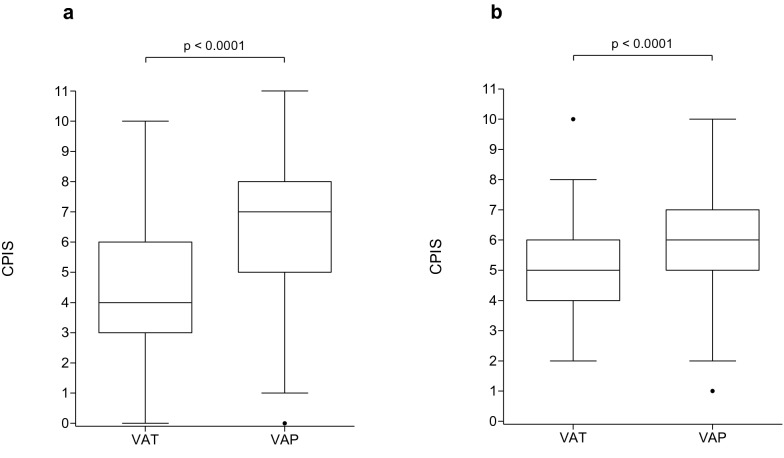
Values of CPIS in patients with VAT and VAP in the derivation (**a**) and validation (**b**) cohorts. Box plot shows the median (horizontal line) and IQR (25th 75th percentile) (box). The whiskers show the lowest data within 1.5 IQR of the lower quartile and highest data within 1.5 IQR of the upper quartile. Data outside 1.5 IQR of the upper or lower quartiles are depicted with a dot. *CPIS* Clinical Pulmonary Infection Score; *VAP* Ventilator-Associated Pneumonia; *VAT* Ventilator-Associated Tracheobronchitis

The distribution of CPIS values was significantly lower in patients with VAT than in those with VAP in the derivation cohort with median values of 4 (3–6) vs 7 (5–8) (*p* < 0.0001) as well as in the validation cohort (5 (4–6) vs 6 (5 –7) (*p* < 0.0001)) (Fig. 1).

**Fig. 2:**
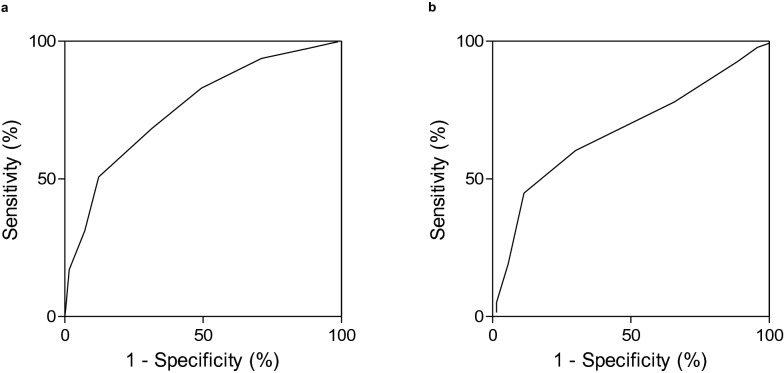
ROC curves of CPIS for the diagnosis of VAP amongst patients with VA LRTI in the derivation (**a**) and validation (**b**) cohorts. A sensitivity analysis was performed for the accuracy of CPIS in diagnosing VAP amongst patients with VA LRTI and ROC curves were computerized in derivation and validation cohorts. *CPIS* Clinical pulmonary infection score, *VAP* Ventilator-associated pneumonia, *VAT* Ventilator-associated tracheobronchitis, *VA LRTI* Ventilator-associated lower respiratory tract infection

ROC analysis showed AUCs at 0.76 (95% CI [0.72–0.79]) in the derivation cohort and 0.67 (95% CI [0.6–0.75]) in the validation cohort (Fig. 2).

**Fig. 3:**
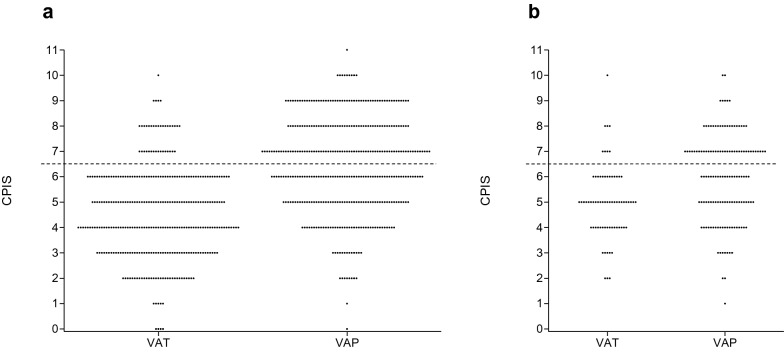
Scatter dot plots of CPIS in patients with VAT and VAP in the derivation (**a**) and validation (**b**) cohorts relatively to the 7-point cut-off. The CPIS value is depicted as a single dot for each patient. Dash lines separate patients with a CPIS value ≥ 7 from those with a CPIS value < 7. *CPIS* Clinical pulmonary infection score, *VAP* Ventilator-associated pneumonia, *VAT* Ventilator-associated tracheobronchitis

Performances of the CPIS for the diagnosis of VAP at different cut-offs are summarized in Table 4. A CPIS value ≥ 7 was associated with the highest Youden index in both cohorts. With this cut-off, sensitivity and specificity were respectively found at 0.51 and 0.88 in the derivation cohort, and at 0.45 and 0.89 in the validation cohort. A CPIS value  ≥ 7 was found in 225 patients of the validation cohort and amongst them, 187 were true VAPs (PPV = 0.83). In the derivation cohort, 69 patients had a CPIS value  ≥ 7 and amongst them 61 were true VAPs (PPV = 0.83) (Fig. 3).

**Table 4:** Performances of CPIS for the diagnosis of VAP in patients with VA-LRTI

	CPIS value	Se	Sp	PPV	NPV	LR+	LR-	Youden index
Derivation cohort	≥ 5	0.83	0.51	0.66	0.72	1.68	0.34	0.34
	≥ 6	0.68	0.69	0.71	0.65	2.17	0.46	0.37
	≥ 7	0.51	0.88	0.83	0.61	4.13	0.56	0.39
Validation cohort	≥ 5	0.78	0.34	0.70	0.44	1.19	0.64	0.12
	≥ 6	0.60	0.70	0.80	0.48	2	0.57	0.3
	≥ 7	0.45	0.89	0.88	0.45	3.92	0.62	0.34

*CPIS* Clinical pulmonary infection score, *Se* Sensitivity, *Sp* Specificity, *PPV* Positive predictive value, *NPV* Negative predictive value, *LR *+ Positive likelihood ratio, *LR*- Negative likelihood ratio

In the derivation cohort, inappropriate antimicrobial therapy was associated with a longer duration of mechanical ventilation in patients with a CPIS value  ≥ 7, whilst no difference between groups was found in subjects with a CPIS value  < 7 (Table 5). Comparisons of clinical outcomes according to the appropriateness of antimicrobial therapy in VAT and VAP in derivation and validation cohorts are shown in Additional file 5.

**Table 5:** Comparisons of clinical outcomes according to the value of CPIS and appropriateness of antimicrobial therapy

	CPIS value	Derivation cohort			Validation cohort		
		Appropriate antimicrobial therapy (*n* = 431)	Inappropriate antimicrobial therapy (*n* = 258)	*P* value	Appropriate antimicrobial therapy (*n* = 95)	Inappropriate antimicrobial therapy (*n* = 111)	*P* value
Days on mechanical ventilation	< 7	14 (8–26)	13 (8–24)	0.59	17.5 (11–32.5)	19 (12–28)	0.82
	≥ 7	14 (8–26)	18 (12–29.5)	*0.03*	16 (11–33)	17 (11–26.5)	0.72
Days in the ICU	< 7	21 (13–31)	21.5 (14–36)	0.37	24 (14.5–36)	25 (14–36.5)	0.76
	≥ 7	19 (13–32)	25 (15–40.5)	0.052	20 (13–30)	20 (15–41)	0.77
ICU mortality	< 7	88 (33%)	65 (34%)	0.93	19 (30%)	23 (32%)	0.82
	≥ 7	62 (38%)	24 (28%)	0.12	7 (23%)	15 (39%)	0.13

Data are presented as number (%) or median (interquartile range)

*ICU* Intensive care unit; *CPIS* Clinical pulmonary infection score

*p* values < 0.05 are indicated in italic characters

## Discussion

In this study, a CPIS value  ≥ 7 appeared as the best cut-off according to the Youden index, with a high specificity and a moderate sensitivity, with similar results in both derivation and validation cohorts.

As far as we know, this is the first study to explore the accuracy of CPIS for the diagnosis of VAP amongst patients with VA-LRTI. CPIS has been widely described as a helpful tool, with a moderate accuracy for the diagnosis of VAP, a best cut-off value of 6 in most studies, and pooled estimates for sensitivity and specificity at 0.65 and 0.64, respectively [9].

The question of early identifying patients who will develop a diagnosis of VAP may be critical, as therapeutic managements of VAT and VAP currently differ. Indeed, current guidelines recommend that VAP should be treated with appropriate antimicrobial therapy, by contrast with VAT for which such a treatment remains not recommended [2, 10]. In that view, considering a CPIS value  ≥ 7 as a criterion for the early initiation of antimicrobial therapy may appear clinically relevant, as this cut-off was associated with good specificities and PPVs > 0.8 in both cohorts. Furthermore, a CPIS value  ≥ 7 was found in 187/369 (51%) and 61/136 (45%) patients with VAPs in our derivation and validation cohorts, respectively, suggesting that this threshold may allow an early diagnosis in almost half cases of VAP. Conversely, we may note the moderate to poor NPV of the CPIS to exclude the diagnosis of VAP. Accordingly, it appears that using the CPIS in subjects with clinical suspicion of VA-LRTI and positive microbiological sampling should mostly be considered for the early initiation of antibiotics in patients with a CPIS value  ≥ 7.

However, these considerations must be taken with caution. First, because appropriate antimicrobial therapy in patients with CPIS ≥ 7 was poorly associated with favourable outcomes in our cohorts, yet this result might be a consequence of the differences in characteristics between patients receiving appropriate and inappropriate antibiotic treatments. Second, the question of initiating antibiotic treatments in patients with VAT remains unclear. Indeed, a continuum between VAT and VAP cannot be ruled out, as a higher risk of pneumonia was reported in patients with VAT in the TAVeM study [1]. In addition, data from this cohort suggest that the risk of progression from VAT to VAP would be reduced in patients receiving antibiotic treatment. On the other hand, current French and international guidelines do not address the initiation of antibiotics in VAT, because of a lack of evidence supporting this strategy [2]. Thus, further investigations are warranted to clarify whether patients with VAT should benefit or not from antimicrobial therapy.

One apparently surprising result of our study was the 25% rate of patients with CPIS values  ≥ 6 in VATs. This might be explained by the fact that our study was restricted to patients with symptoms of lower respiratory tract infections. This is likely to have resulted in overall greater CPIS values in our population than usually reported, especially in patients without VAP [9]. As a consequence, a more intense clinical presentation seems required to accurately segregate VAPs from VATs than to distinguish VAPs from patients without respiratory infections. This might partly explain the optimal cut-off found at 7 in our study, which is higher than the 6 points threshold usually reported in the literature [9].

In contrast, we report CPIS values found at 0 or 1 in several patients with VAPs and VATs in both cohorts. This result is actually reflecting the uncomplete clinical presentations initially observed in some patients. In these, the symptoms could initially include a mild increase of body temperature without reaching 38.5 °C, the appearance of few tracheal secretions, or a moderate worsening of the PaO_2_/FiO_2_ ratio without falling below 240, thus motivating the respiratory microbiological sampling, although the CPIS was still calculated at 0 or 1 by then. However, all these patients eventually reached the criteria for VA-LRTI, with a worsening of their symptoms, thus completing their clinical presentation. This frequent progression at the initial stage of VA-LRTI raises the question of whether the calculation of CPIS at a single time-point can give enough information to distinguish VAP from VAT. The evaluation of the relationship between CPIS calculated at the time of clinical suspicion of VA-LRTI and variations of CPIS over 24 h before that time-point (Delta CPIS) in our derivation cohort brings some interesting insights about that question. This analysis reveals that nearly all patients with a CPIS value ≥ 7 also had a Delta CPIS ≥ 0 and that CPIS was subsequently correlated to Delta CPIS in VAT and in VAP (Additional file 6). Furthermore, Delta CPIS does not seem to show greater accuracy than CPIS to differentiate VAT from VAP (Additional file 7). These results convey the idea that subjects with greater CPIS are not only exhibiting marked features of respiratory infection, but are also worsening their symptoms, thus supporting the reliability of the CPIS measured at a single time-point to discriminate VATs from VAPs.

Other parameters than the CPIS might be proposed to improve the early diagnosis of VAP. VAP seemed to be associated with a higher mortality in ICU than VAT, yet this result was not statistically significant in the validation cohort. This result suggests that a diagnosis of VAP might be associated with more severe organ failures, and especially with more severe levels of hypoxaemia. This hypothesis is supported by the results of the TAVeM study, showing that significantly more patients with VAP had an episode of worsening gas exchange than did those with VAT [1]. Therefore, severity scores like SOFA, calculated at the time of the clinical suspicion of VA-LRTI, could help to improve the early detection of VAP, particularly through evaluation of the respiratory SOFA. A similar strategy has already been developed through the Infection Probability Score, although this score does not apply specifically to VAP but to nosocomial infections in general [11]. Furthermore, such a strategy has never been evaluated specifically for VA-LRTI.

Routine biomarkers like CRP and PCT have been evaluated for the identification of VAP, with variable diagnostic performances. Luyt et al. reported a poor accuracy of PCT for the diagnosis of VAP, with ROC AUCs respectively found at 0.51 and 0.62 for D-1 PCT and PCT increase. To be noted that adjunction of PCT did not significantly improve the accuracy of the CPIS for the diagnosis of VAP in this study, with an AUC rising from 0.68 for CPIS alone to 0.69 for CPIS combined with PCT [12]. In another report, Charles et al. showed good performances of PCT variations for the diagnosis of nosocomial infections, with AUCs > 0.8 in ROC analysis and similar results for the diagnosis of VAP. In this work, PCT variations were above all associated with a good specificity and high PPVs [13]. On the other hand, Povoa et al. showed a moderate performance of CRP for the diagnosis of VAP, with AUCs > 0.7 for variations of CRP in their ROC analysis [14]. Furthermore, at the day of VAP diagnosis, they observed that a single measurement of CRP was useful in particular for the exclusion of VAP diagnosis. Beside these routine biomarkers, some authors have investigated the potential usefulness of sTREM-1, yet with disappointing results showing very poor accuracy for the diagnosis of VAP [15, 16].

These data underline the potential utility of these biomarkers in differentiating VAT from VAP. However, their moderate accuracy when taken as single parameter highlights the hypothetical interest of a mixed evaluation integrating one or several of these variables in a CPIS-based score.

The distinction between VAT and VAP currently relies on the presence of at least one new infiltrate on the chest radiography. However, the relevance of this criterion remains questionable, given its low accuracy in diagnosing microbiologically confirmed VAP amongst critically ill subjects undergoing mechanical ventilation [3]. This inaccuracy mainly results from the difficulties to distinguish infiltrates of infectious cause from other aetiologies, like pleural effusions, cardiac overload or atelectasis, which are frequently present in ICU patients [4]. In that view, lung ultrasound has been proposed as a promising tool for the diagnosis of VAP, and could thus be considered to distinguish VAT from VAP, yet its utility remains limited because of its poor inter-operator reproducibility [17]. Computed tomography (CT) could also be proposed to enhance the detection of lung infiltrates. Indeed, Self et al. reported that only 43.5% of the patients presenting to ED with opacities on CT had images of pneumonia detected on chest radiography [18]. Therefore, CT could allow a better differentiation between VAT and VAP. However, its greater cost, associated with the necessity to transport patients, and higher exposure to radiations limit its use in routine clinical practice. All these tools could improve the accuracy of the CPIS and should probably be assessed in a future updated version of this score.

Our study has several limitations. First, this was a retrospective analysis, thus limiting the parameters that could be studied in our cohorts. Above all, studying the accuracy of inflammation biomarkers like CRP or PCT would have been interesting, but could not be performed because these parameters were not measured in a sufficient number of patients in our validation cohort. Second, this study was performed in patients with microbiologically confirmed VA-LRTI, limiting the applicability of our results at the patient’s bedside, because of the frequent delay between processing of the microbiological sampling and culture positivity. Third, evaluating the CPIS to distinguish VAT from VAP might appear questionable in our study, as the radiographic criterion is part of the items required for the calculation of this score. Nevertheless, it must be noted that the CPIS was calculated once for all at the time of microbiological sampling in the respiratory tract, which corresponded to the moment of the clinical suspicion of VA-LRTI. This is to oppose the methods used to retrospectively classify patients between VAT and VAP, with all the biological, clinical and radiological data available to make a final diagnosis. This last point must be considered regarding the frequency of uncomplete initial presentation with initial absence of radiographic signs, representing more than 60% of VAP in the study performed by Ramirez et al. [5]. Fourth, the population of our study included a lower percentage of surgical admissions in the validation cohort than in the derivation cohort. This might have interfered with our results, as a poor accuracy of CPIS has been reported in surgical patients, because non-infectious causes of lung injury may represent a confounder in this population [19, 20]. Fifth, the rates of appropriate initial antibiotic treatments were significantly different between VAPs and VATs in both cohorts. Accordingly, we could not draw any conclusion regarding the benefit of appropriate antimicrobial therapy in patients with a CPIS value ≥ 7. Sixth, ROC analysis showed a lower AUC of CPIS in our validation cohort, thus underlining a weaker overall accuracy in this population. Finally, lung infiltrates were probably missed in some patients with VAP, given the reported lack of sensitivity of chest radiography for the detection of pneumonia [3, 4]. However, the use of chest radiography allowed us to diagnose VAT and VAP using the same criteria than in the TAVeM study. Accordingly, our definitions of VAP and VAT are consistent with those in which a difference in mortality rates was reported [1]. Further explorations in a larger prospective multicentre study are needed to confirm our findings.

## Conclusions

In patients with evidence of VA-LRTI, a CPIS value  ≥ 7 allowed the early diagnosis of half cases of VAP with specificity and PPV above 80% in our derivation and validation cohorts. The CPIS might thus be considered as a helpful tool to drive the early initiation of antimicrobial therapy in patients with VA-LRTI.

## Supplementary information

**Additional file 1:** Description of the clinical pulmonary infection score.

**Additional file 2:** Comparison of baseline characteristics in derivation and validation cohorts.

**Additional file 3:** Comparison of clinical outcomes in derivation and validation cohorts.

**Additional file 4:** Comparison of microbiological findings in derivation and validation cohorts.

**Additional file 5:** Comparison of clinical outcomes according to the appropriateness of antimicrobial therapy in VAT and VAP in derivation and validation cohorts.

**Additional file 6:** Relationship between Delta CPIS and CPIS at the time of microbiological sampling in patients with VAT and VAP, respectively, in the derivation cohort. **a** Dot plots of Delta CPIS and CPIS value at the time of microbiological sampling. **b** Scatter plots of Delta CPIS vs CPIS at the time of microbiological sampling. Spearman’s correlation tests were performed for VATs (*r*_*s*_ = 0.42 (95% CI 0.32 –0.51), *p* < 10^−3^) and VAPs (*r*_*s*_ = 0.39 (95% CI 0.3–0.48), *p* < 10^−3^). Delta CPIS was calculated as the difference between CPIS value 24 h before microbiological sampling and CPIS value at the time of microbiological sampling. Data for calculation of Delta CPIS were not available in the validation cohort. *CPIS* Clinical pulmonary infection score, *VAP* Ventilator-associated pneumonia, *VAT* Ventilator-associated tracheobronchitis.

**Additional file 7:** Performances of Delta CPIS for the diagnosis of VAP in patients with VA LRTI in the derivation cohort.

## Data Availability

The datasets used and analyzed during the current study are available from the corresponding author on reasonable request.

## Abbreviations

AUC: Area under the curve
CPIS: Clinical pulmonary infection score
CT: Computed tomography
ICU: Intensive care unit
NPV: Negative predictive value
PPV: Positive predictive value
ROC: Receiver operating characteristics
VA-LRTI: Ventilator-associated lower respiratory tract infections
VAP: Ventilator-associated pneumonia
VAT: Ventilator-associated tracheobronchitis
