# Supplementary material for: Accuracy of the clinical pulmonary infection score to differentiate ventilator-associated tracheobronchitis from ventilator-associated pneumonia
Source: Ann Intensive Care. 2020 Aug 3;10:101. doi: 10.1186/s13613-020-00721-4 (PMC7396887; doi:10.1186/s13613-020-00721-4)
Supplement: Supplementary file 1 — Additional file 1: Description of the clinical pulmonary infection score. [file 13613_2020_721_MOESM1_ESM.doc]

**Additional file 1. Description of the Clinical Pulmonary Infection Score**

| Component | Value | Points |
| --- | --- | --- |
| Temperature (°C) | ≥ 36.5 and ≤ 38.4 | 0 |
|  | ≥ 38.5 and ≤ 38.9 | 1 |
|  | ≥ 39.0 and ≤ 36.0 | 2 |
| Blood leukocytes (/mm^3^) | ≥ 4000 and ≤ 11,000  < 4000 or > 11,000 | 0  1 |
| Tracheal secretions | Few  Moderate  Large  Purulent | 0  1  2  +1 |
| Oxygenation PaO2/FiO2 (mmHg) | > 240 or presence of ARDS  ≤ 240 and absence of ARDS | 0  2 |
| Chest radiograph | No infiltrate  Patchy or diffuse infiltrate  Localized infiltrate | 0  1  2 |

From Luna *et al[[1]](#footnote-2)*. *ARDS* Acute Respiratory Distress Syndrome.

1. Carlos M. Luna and others, ‘Resolution of Ventilator-Associated Pneumonia: Prospective Evaluation of the Clinical Pulmonary Infection Score as an Early Clinical Predictor of Outcome’*, Critical Care Medici*ne, 31.3 (2003), 676–82 <https://doi.org/10.1097/01.CCM.0000055380.86458.1E>. [↑](#footnote-ref-2)
