# Supplementary material for: Accuracy of the clinical pulmonary infection score to differentiate ventilator-associated tracheobronchitis from ventilator-associated pneumonia
Source: Ann Intensive Care. 2020 Aug 3;10:101. doi: 10.1186/s13613-020-00721-4 (PMC7396887; doi:10.1186/s13613-020-00721-4)
Supplement: Supplementary file 2 — Additional file 2: Comparison of baseline characteristics in derivation and validation cohorts. [file 13613_2020_721_MOESM2_ESM.doc]

**Additional file 2. Comparison of baseline characteristics in derivation and validation cohorts**

|  | Derivation cohort (n=689) | Validation cohort (n=206) | p value |
| --- | --- | --- | --- |
| Sex |  |  |  |
| Male | 463 (67%) | 153 (74%) |  |
| Female | 226 (33%) | 53 (26%) | 0.054 |
| Age (years) | 59.35 (17.54) | 55.07 (16.23) | **0.002** |
| SAPS II | 49.4 (17.95) | 58.15 (17.81) | **< 10^-3^** |
| SOFA | 7.87 (3.71) | 8.32 (4.21) | 0.14 |
| Admission category |  |  |  |
| Medical | 393 (57%) | 174 (84%) |  |
| Surgical | 296 (43%) | 32 (16%) | **< 0.001** |
| COPD | 125 (18%) | 27 (13%) | 0.09 |
| Diabetes mellitus | 135 (20%) | 40 (19%) | 0.96 |
| Immunocompromised patients | 55 (8%) | 30 (15%) | **0.005** |
| Chronic heart failure | 46 (7%) | 32 (16%) | **< 0.001** |
| Chronic respiratory failure | 62 (9%) | 10 (5%) | 0.055 |
| Cirrhosis | 31 (4%) | 13 (6%) | 0.29 |
| Previous antibiotic use | 442 (64%) | 167 (81%) | **< 0.001** |

Data are presented as number (%) or mean (SD). p values < 0.05 are indicated in bold. *COPD* Chronic Obstructive Pulmonary Disease; *SAPS* Simplified Acute Physiology Score; *SOFA* Sequential Organ Failure Assessment; *VAP* Ventilator Associated Pneumonia; *VAT* Ventilator Associated Tracheobronchitis.
