# Supplementary material for: Accuracy of the clinical pulmonary infection score to differentiate ventilator-associated tracheobronchitis from ventilator-associated pneumonia
Source: Ann Intensive Care. 2020 Aug 3;10:101. doi: 10.1186/s13613-020-00721-4 (PMC7396887; doi:10.1186/s13613-020-00721-4)
Supplement: Supplementary file 3 — Additional file 3: Comparison of clinical outcomes in derivation and validation cohorts. [file 13613_2020_721_MOESM3_ESM.doc]

**Additional file 3. Comparison of clinical outcomes in derivation and validation cohorts**

|  | Derivation cohort (n = 689) | Validation cohort (n = 206) | p value |
| --- | --- | --- | --- |
| Days on mechanical ventilation | 13 (8 – 25) | 17 (12 – 28) | **< 0.001** |
| Days in the ICU | 21 (14 – 33) | 23 (14 – 36) | 0.18 |
| ICU mortality | 239 (35%) | 64 (31%) | 0.34 |

Data are presented as number (%) or median (interquartile range). p values < 0.05 are indicated in bold*. ICU* Intensive Care Unit; *VAP* Ventilator Associated Pneumonia; *VAT* Ventilator Associated Tracheobronchitis.
