# Supplementary material for: Accuracy of the clinical pulmonary infection score to differentiate ventilator-associated tracheobronchitis from ventilator-associated pneumonia
Source: Ann Intensive Care. 2020 Aug 3;10:101. doi: 10.1186/s13613-020-00721-4 (PMC7396887; doi:10.1186/s13613-020-00721-4)
Supplement: Supplementary file 4 — Additional file 4: Comparison of microbiological findings in derivation and validation cohorts. [file 13613_2020_721_MOESM4_ESM.doc]

**Additional file 4. Comparison of microbiological findings in derivation and validation cohorts**

|  | Derivation cohort (n=689) | Validation cohort (n=206) | p value |
| --- | --- | --- | --- |
| *Streptococcus pneumoniae* | 40 (6%) | 7 (3%) | 0.17 |
| *Stenotrophomonas maltophila* | 31 (4%) | 10 (5%) | 0.83 |
| MRSA | 16 (2%) | 2 (1%) | 0.39 |
| MSSA | 146 (21%) | 27 (13%) | **0.01** |
| *Serratia marcescens* | 28 (4%) | 8 (4%) | 0.91 |
| *Pseudomonas aeruginosa* | 168 (24%) | 49 (24%) | 0.86 |
| *Proteus mirabilis* | 29 (4%) | 8 (4%) | 0.84 |
| *Klebsiella pneumoniae* | 101 (15%) | 39 (19%) | 0.14 |
| *Haemophilus influenzae* | 57 (8%) | 10 (5%) | 0.1 |
| *Escherichia coli* | 77 (11%) | 14 (7%) | 0.068 |
| *Enterobacter* spp | 81 (12%) | 25 (12%) | 0.88 |
| *Citrobacter freundii* | 13 (2%) | 5 (2%) | 0.58 |
| *Acinetobacter baumannii* | 41 (6%) | 2 (1%) | **0.001** |

Data are presented as number (%) or mean (SD). p values < 0.05 are indicated in bold*. COPD* Chronic Obstructive Pulmonary Disease; *MSSA* Methicillin Sensitive Staphylococcus Aureus; *MRSA* Methicillin Resistant Staphylococcus Aureus; *SAPS* Simplified Acute Physiology Score; *SOFA* Sequential Organ Failure Assessment; *VAP* Ventilator Associated Pneumonia; *VAT* Ventilator Associated Tracheobronchitis.
