# Supplementary material for: Accuracy of the clinical pulmonary infection score to differentiate ventilator-associated tracheobronchitis from ventilator-associated pneumonia
Source: Ann Intensive Care. 2020 Aug 3;10:101. doi: 10.1186/s13613-020-00721-4 (PMC7396887; doi:10.1186/s13613-020-00721-4)
Supplement: Supplementary file 5 — Additional file 5: Comparison of clinical outcomes according to the appropriateness of antimicrobial therapy in VAT and VAP in derivation and validation cohorts. [file 13613_2020_721_MOESM5_ESM.doc]

**Additional file 5. Comparison of clinical outcomes according to the appropriateness of antimicrobial therapy in VATs and VAPs in derivation and validation cohorts**

|  | VA-LRTI category | Derivation cohort | | | Validation cohort | | |
| --- | --- | --- | --- | --- | --- | --- | --- |
|  |  | Appropriate  antimicrobial therapy  (n = 431) | Inappropriate  antimicrobial therapy  (n = 258) | p value | Appropriate  antimicrobial therapy  (n = 95) | Inappropriate  antimicrobial therapy  (n = 111) | p value |
| Days on mechanical ventilation | VAT | 14.5 (8 – 22) | 13 (9 – 24) | 0.95 | 18 (13.5 – 30.5) | 18.5 (13 – 29) | 0.89 |
|  | VAP | 14 (8 – 25) | 18 (10 – 28.5) | **0.044** | 16 (10 – 33) | 17 (12 – 26) | 0.96 |
|  | | | | |  | | |
| Days in the ICU | VAT | 22 (15 – 32) | 20.5 (14 – 35) | 0.74 | 24 (18 – 33.5) | 28 (19 – 44) | 0.32 |
|  | VAP | 19 (12 – 31) | 25 (15 – 40) | **< 0.001** | 20 (13 – 35) | 22 (14 – 34.5) | 0.94 |
|  | | | | |  | | |
| ICU mortality | VAT | 47 (27%) | 46 (32%) | 0.38 | 11 (27%) | 7 (23%) | 0.69 |
|  | VAP | 103 (40%) | 43 (38%) | 0.76 | 15 (27%) | 31 (38%) | 0.18 |

Data are presented as number (%) or median (interquartile range). p values < 0.05 are indicated in bold characters. *ICU* Intensive Care Unit; *CPIS* Clinical Pulmonary Infection Score.
