# Supplementary figures and images for: Accuracy of the clinical pulmonary infection score to differentiate ventilator-associated tracheobronchitis from ventilator-associated pneumonia
Source: Ann Intensive Care. 2020 Aug 3;10:101. doi: 10.1186/s13613-020-00721-4 (PMC7396887; doi:10.1186/s13613-020-00721-4)

Additional file 6

a)

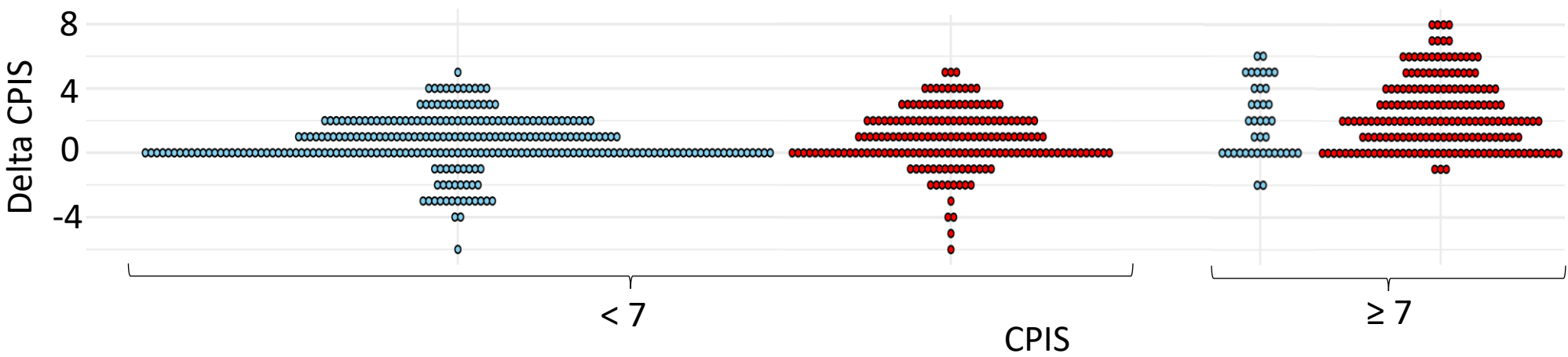

b)

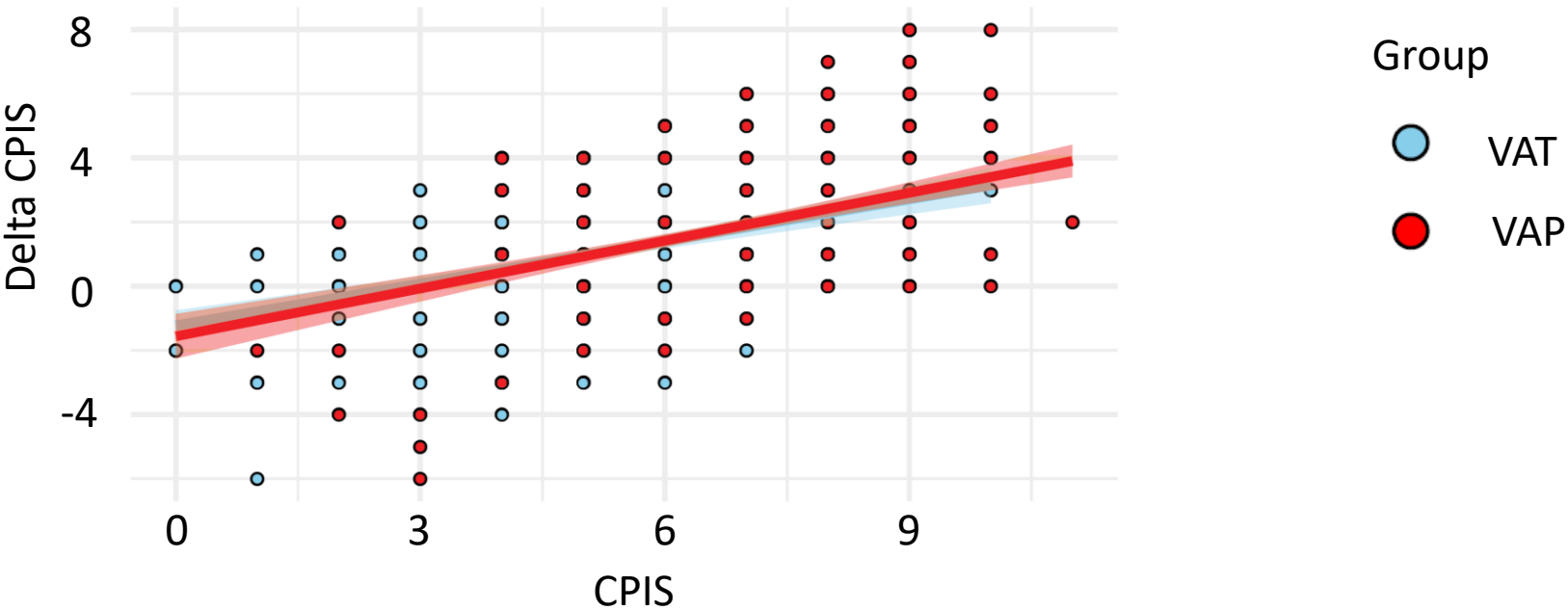

Supplement: Supplementary file 6 — Additional file 6: Relationship between Delta CPIS and CPIS at the time of microbiological sampling in patients with VAT and VAP, respectively, in the derivation cohort. a Dot plots of Delta CPIS and CPIS value at the time of microbiological sampling. b Scatter plots of Delta CPIS vs CPIS at the time of microbiological sampling. Spearman’s correlation tests were performed for VATs (rs = 0.42 (95% CI 0.32 –0.51), p < 10−3) and VAPs (rs = 0.39 (95% CI 0.3–0.48), p < 10−3). Delta CPIS was calculated as the difference between CPIS value 24 h before microbiological sampling and CPIS value at the time of microbiological sampling. Data for calculation of Delta CPIS were not available in the validation cohort. CPIS Clinical pulmonary infection score, VAP Ventilator-associated pneumonia, VAT Ventilator-associated tracheobronchitis. [file 13613_2020_721_MOESM6_ESM.pdf]
