# Supplementary material for: Accuracy of the clinical pulmonary infection score to differentiate ventilator-associated tracheobronchitis from ventilator-associated pneumonia
Source: Ann Intensive Care. 2020 Aug 3;10:101. doi: 10.1186/s13613-020-00721-4 (PMC7396887; doi:10.1186/s13613-020-00721-4)
Supplement: Supplementary file 7 — Additional file 7: Performances of Delta CPIS for the diagnosis of VAP in patients with VA LRTI in the derivation cohort. [file 13613_2020_721_MOESM7_ESM.doc]

**Additional file 7. Performances of Delta CPIS for the diagnosis of VAP in patients with VA LRTI in the derivation cohort**

|  | Delta  CPIS | Se | Sp | PPV | NPV | LR+ | LR- | Youden index |
| --- | --- | --- | --- | --- | --- | --- | --- | --- |
| Derivation cohort | ≥ 1 | 0.64 | 0.51 | 0.60 | 0.55 | 1.31 | 0.70 | 0.15 |
|  | ≥ 2 | 0.48 | 0.70 | 0.65 | 0.54 | 1.59 | 0.75 | 0.18 |
|  | ≥ 3 | 0.30 | 0.87 | 0.72 | 0.52 | 2.25 | 0.81 | 0.17 |

Delta CPIS was calculated as the difference between CPIS value 24h before microbiological sampling and CPIS value at the time of microbiological sampling. Data for calculation of Delta CPIS were not available in the validation cohort. *CPIS* Clinical Pulmonary Infection Score; *Se* Sensitivity; *Sp* Specificity; *PPV* Positive Predictive Value; *NPV* Negative Predictive Value; *LR+* Positive Likelihood Ratio; *LR-* Negative Likelihood Ratio
